# Removal of BFL-1 sensitises some melanoma cells to killing by BH3 mimetic drugs

**DOI:** 10.1038/s41419-022-04776-y

**Published:** 2022-04-04

**Authors:** Lahiru Gangoda, Robyn L. Schenk, Lin Tai, Pacman Szeto, Jen G. Cheung, Andreas Strasser, Guillaume Lessene, Mark Shackleton, Marco J. Herold

**Affiliations:** 1 The Walter and Eliza Hall Institute of Medical Research (WEHI), 1G Royal Parade, Parkville, Melbourne, VIC 3052 Australia; 2 Department of Medical Biology, University of Melbourne, Parkville, Melbourne, VIC 3052 Australia; 3 Department of Oncology, Alfred Health, Melbourne, VIC 3004 Australia; 4 Central Clinical School, Faculty of Medicine, Nursing and Allied Health, Monash University, Melbourne, VIC Australia; 5 Department of Pharmacology and Therapeutics, University of Melbourne, Parkville, Melbourne, VIC 3052 Australia

**Keywords:** Cancer, Cell biology

Metastatic melanoma is an aggressive form of skin cancer with <20% 5-year survival rate when detected at advanced stages [1]. *BRAF* mutations occur in 60% while NRAS mutations occur in 20% of melanoma patients both causing constitutive activation of the MAPK pathway, thereby driving uncontrolled cell proliferation and increasing resistance to cell death [2]. In patients with melanoma, the activated BRAF-mutated kinase can be inhibited by BRAF-targeting drugs, and its downstream protein mitogen-activated protein kinase kinase (MEK) can be inhibited by a MEK-targeting drug [3]. However, there is no targeted drug for mutant NRAS, hence in the case of NRAS mutant melanomas the current approaches are largely concentrated on downstream signalling pathways such as using MEK-targeted inhibitors [4]. Yet, nearly all melanoma patients eventually relapse. Hence, improved therapies for patients with melanomas are urgently required. One such approach could be to use cell death-inducing BH3 mimetic drugs that inhibit the pro-survival proteins of the BCL-2 family (BCL-2, BCL-XL, BCL-W, MCL-1 and BFL-1) [5, 6]. So far, there are no publications describing BH3 mimetic drugs targeting the pro-survival protein BFL-1, whose gene is frequently amplified and whose mRNA is highly expressed in melanoma [7–9], We revealed by western blot analysis that BFL-1 protein can be readily detected in >50% of human melanoma-derived cell lines. BCL-XL and MCL-1 could also be detected (Supplementary Fig. 1). It has previously been shown that treating some of these cell lines with the BCL-2 inhibitor ABT-199 alone or in combination with the mutant BRAF inhibitor does not kill the cells [10]. In addition, we were able to detect BFL-1 protein in several human melanoma-derived xenograft samples (Supplementary Fig. 2).

We treated several melanoma-derived cell lines for 72 h with the MCL-1 inhibitor S63845 [6], the BCL-XL inhibitor A1331852 [11] or the BRAF^V600E^ inhibitor PLX4032 [12]. While the MCL-1 inhibitor efficiently killed UACC257, SKMEL2 and HMCB cell lines, the BCL-XL inhibitor or the mutant BRAF inhibitor had almost no effect on the survival of all cell lines tested (Supplementary Fig. 3a, b). Reduction of BFL-1 by RNA interference was reported to lead to spontaneous killing and to enhanced sensitivity to 5-fluorouracil in melanoma cells [13]. To test the role of BFL-1 in sensitivity of melanoma cells to BH3-mimetic drugs, we deleted *BCL2A1* in three BRAF mutant melanoma cells (M14 and UACC257 show high BFL-1 expression; LOXIMVI show medium BFL-1 expression) and one NRAS mutant cell (SKMEL30 show high BFL-1 expression) by using our inducible CRISPR/Cas9 platform [14]. Western blot analysis of the CRISPR/Cas9 engineered melanoma cells confirmed loss of the BFL-1 protein (Supplementary Fig. 4a). BFL-1 deletion did not increase the spontaneous death of these melanoma cells (Supplementary Fig. 4b), nor their sensitivity to single-agent treatment with any of the BH3-mimetic drugs tested or PLX4032, when used as single agents (Fig. 1a–d). Of note, removal of BFL-1 increased the death of M14 and SKMEL30 melanoma cells treated with a combination of the MCL-1 and the BCL-XL inhibitors (Fig. 1a, b). The removal of BFL-1 had no additional impact on the response of the UACC257 and LOXIMVI melanoma cells to any of the drug combinations tested (Fig. 1c, d).

**Fig. 1:**
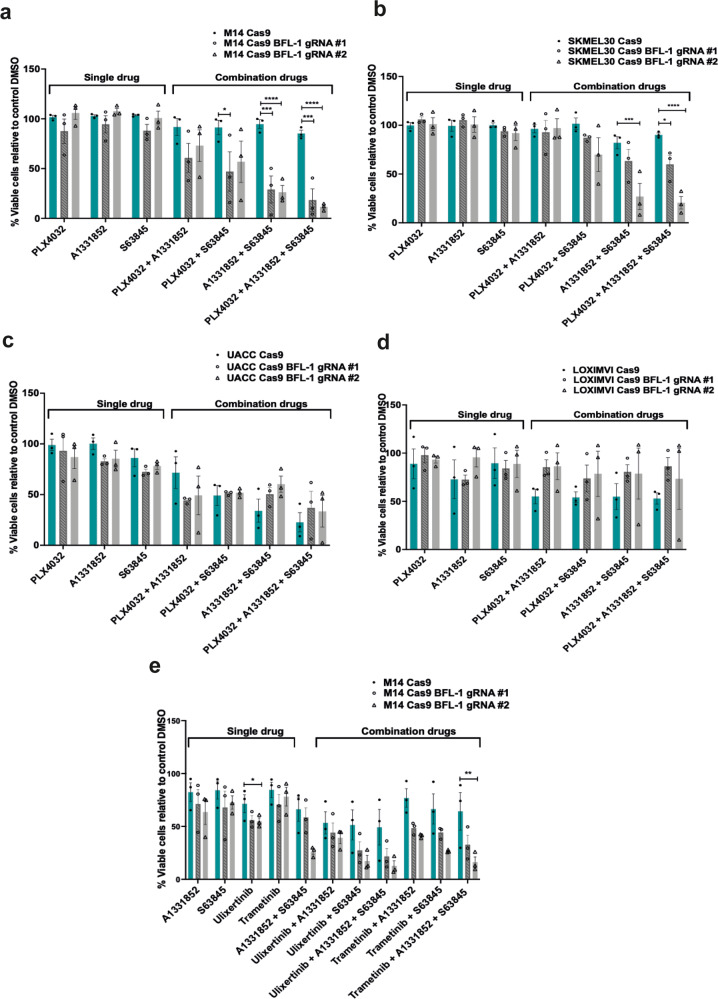
Testing the responses of diverse parental and BFL-1 knockout human melanoma cell lines to different drug regimens. The survival of parental and BFL-1 knockout melanoma cells was determined by FACS analysis after staining with Annexin V-AF647 and DAPI after 72 h of treatment with the indicated drugs at the indicated concentrations. **a**–**d** 1 μM MCL-1 inhibitor S63845, 1 μM BCL-XL inhibitor A1331852 and 1 μM BRAF inhibitor PLX4032 were used alone or in combination. **e** 1 μM MCL-1 inhibitor S63845, 1 μM BCL-XL inhibitor A1331852, 0.5 μM ERK1/2 inhibitor Ulixertinib and 5 nM MEK1/2 inhibitor Trametinib were used alone or in combination. Data represent mean ± SEM of three independent experiments. *P* values were calculated by performing two-way ANOVA followed by multiple comparisons testing. *P < 0.05, **P < 0.01, ***P < 0.001, ***P < 0.0001. DMSO was used as the vehicle control.

Since the loss of BFL-1 increased the killing of M14 melanoma cells when combined with BH3 mimetic drugs that target MCL-1 and BCL-XL, we next tested the response of these cells to combination treatments that also include an inhibitor of MEK1/2, trametinib, or an inhibitor of ERK, ulixertinib, respectively (Fig. 1e). These inhibitors target the constitutively activated MAPK pathway in *BRAF* mutant melanomas. Combined inhibition of MEK1/2, MCL-1 and BCL-XL resulted in the stronger killing of BFL-1 knockout cells compared to the parental cells. No increase in cell killing was observed with ulixertinib, comparing the parental to the BFL-1 knockout M14 melanoma cells.

Our findings identified BFL-1 as a factor that mediates resistance to combined MCL-1 and BCL-XL inhibition in certain melanoma cells. However, out of the four cell lines texted this was mostly evident in M14 cells, suggesting that their survival is safeguarded by three pro-survival BCL-2 proteins, BFL-1, MCL-1 and BCL-XL. Thus, to achieve efficient killing of these malignant cells, all three of these pro-survival proteins need to be inhibited. This could be achieved either by combinations of BH3 mimetic drugs or via additional anti-cancer agents (e.g. inhibitors of MEK1/2) that cause up-regulation of pro-apoptotic BH3-only proteins that can neutralise the pro-survival BCL-2 protein(s) that is/are not targeted by the BH3 mimetic drugs [15].

## Supplementary information

Uncropped Western Blots

Supplemental Material

Point by Point Reply

## Data Availability

All data needed to evaluate the conclusions in the paper are present in the paper. Additional data related to this paper may be requested from the corresponding author.
