# Supplementary material for: Removal of BFL-1 sensitises some melanoma cells to killing by BH3 mimetic drugs
Source: Cell Death Dis. 2022 Apr 4;13(4):301. doi: 10.1038/s41419-022-04776-y (PMC8980089; doi:10.1038/s41419-022-04776-y)
Supplement: Supplementary file 1 — Uncropped Western Blots [file 41419_2022_4776_MOESM1_ESM.docx]

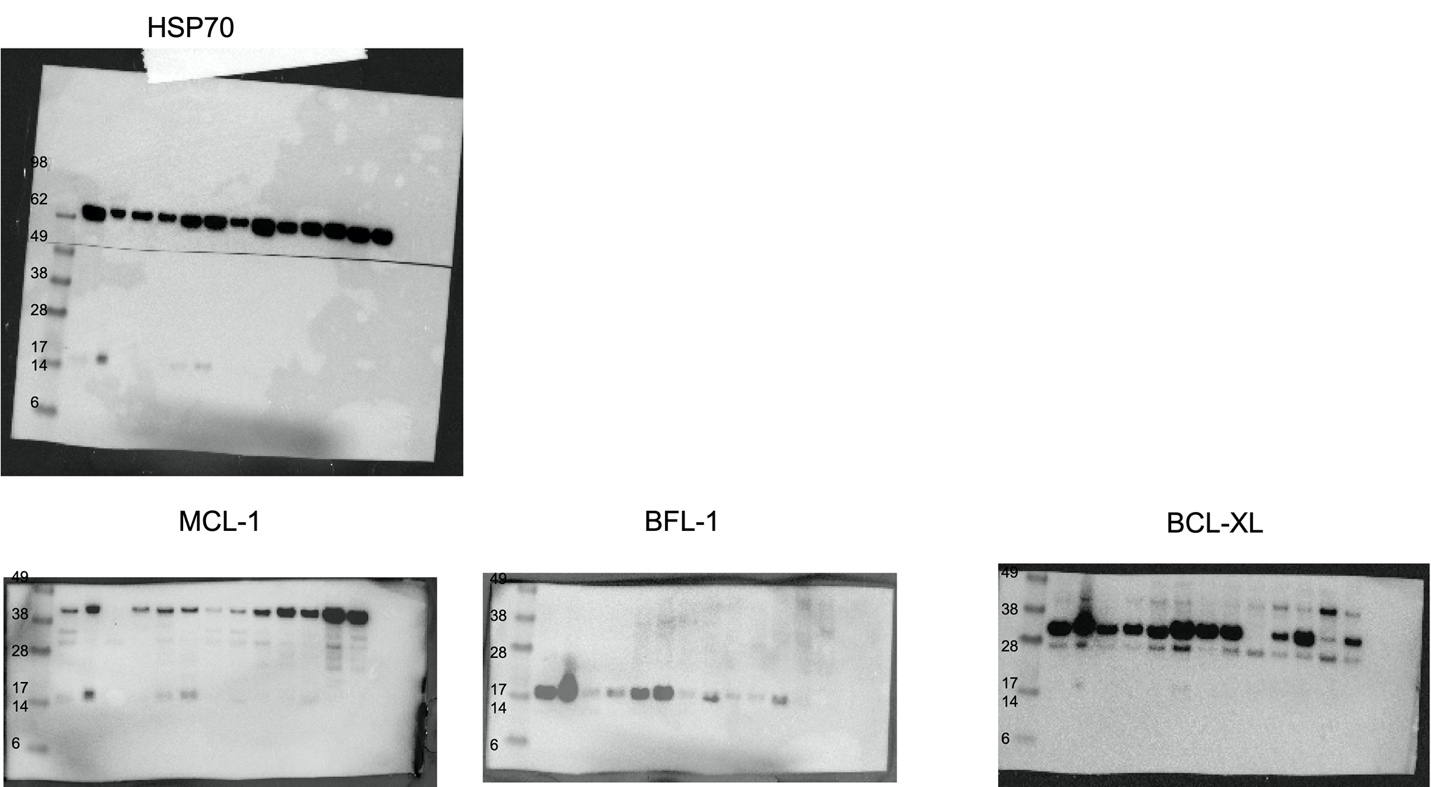


**Supplementary Figure 1. Expression of pro-survival BCL-2 family proteins in melanoma cell lines.**


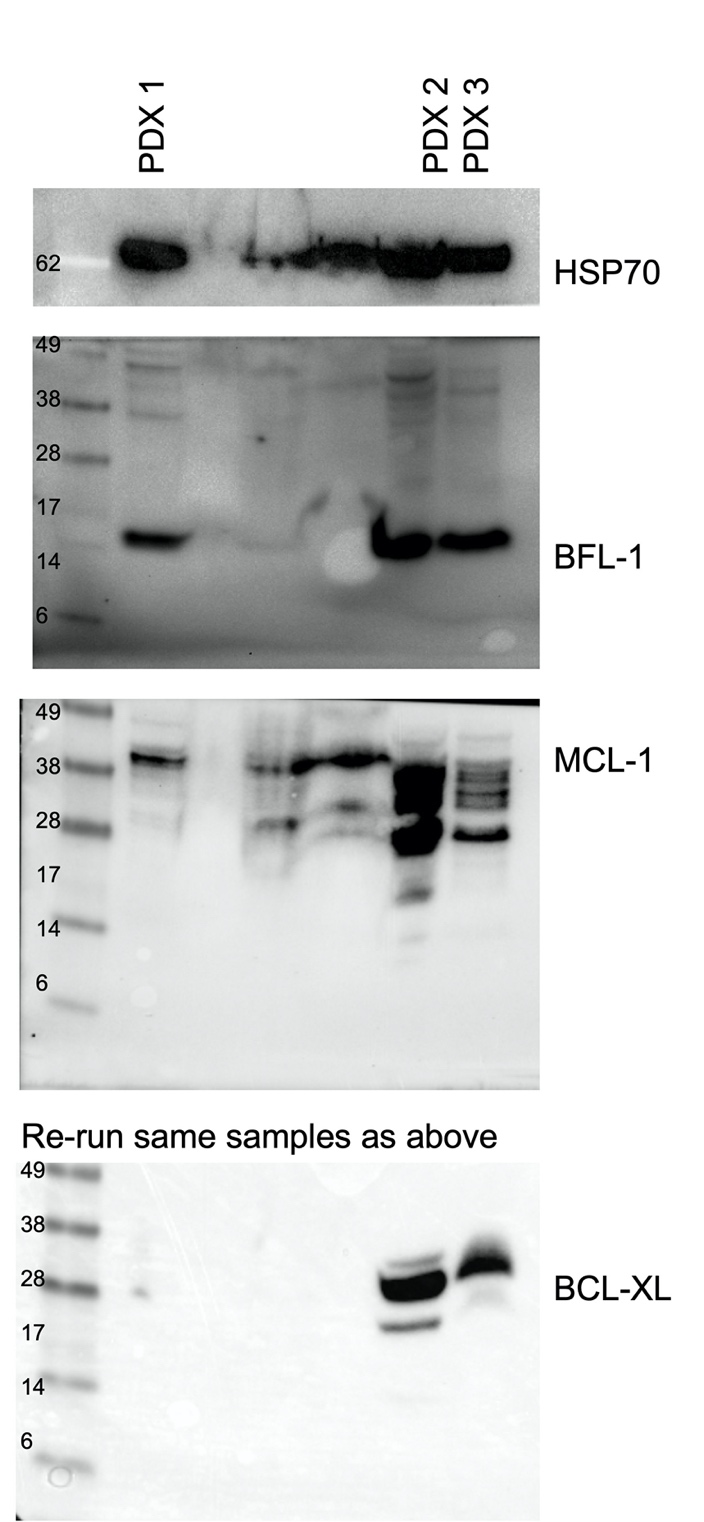


**Supplementary Figure 2. Expression of pro-survival proteins in human melanoma patient derived xenografts (PDX).**

UACC257 SKMEL30
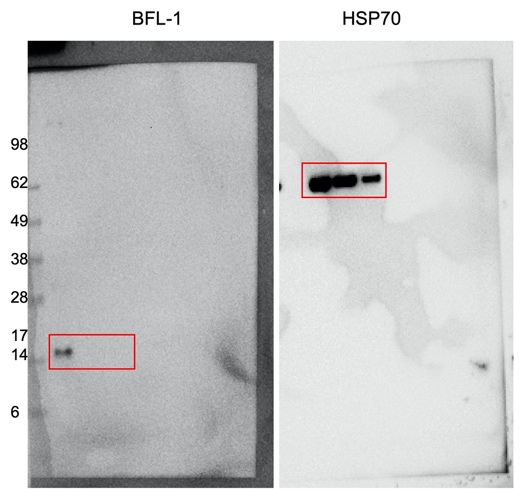

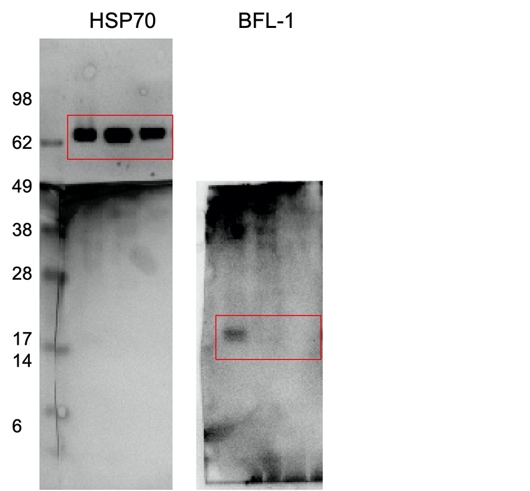


M14 LOXIMVI


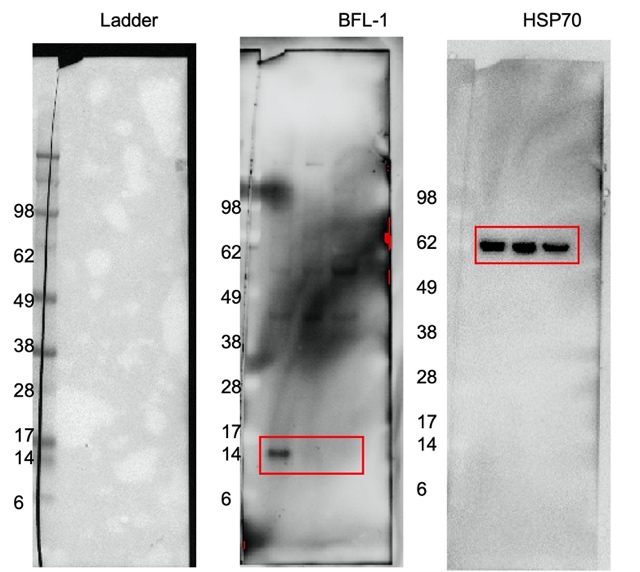

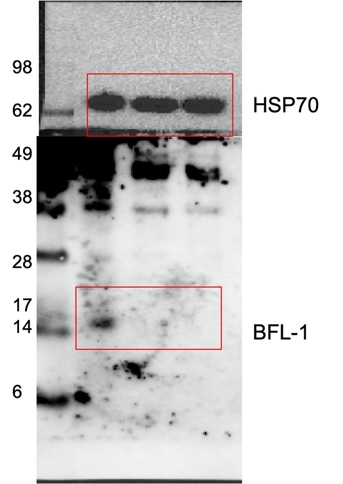


**Supplementary Figure 4.** **Generation of BFL-1 knockout human melanoma cell lines using CRISPR/Cas9 and testing of spontaneous cell death.**
