# Supplementary material for: Removal of BFL-1 sensitises some melanoma cells to killing by BH3 mimetic drugs
Source: Cell Death Dis. 2022 Apr 4;13(4):301. doi: 10.1038/s41419-022-04776-y (PMC8980089; doi:10.1038/s41419-022-04776-y)
Supplement: Supplementary file 2 — Supplemental Material [file 41419_2022_4776_MOESM2_ESM.docx]

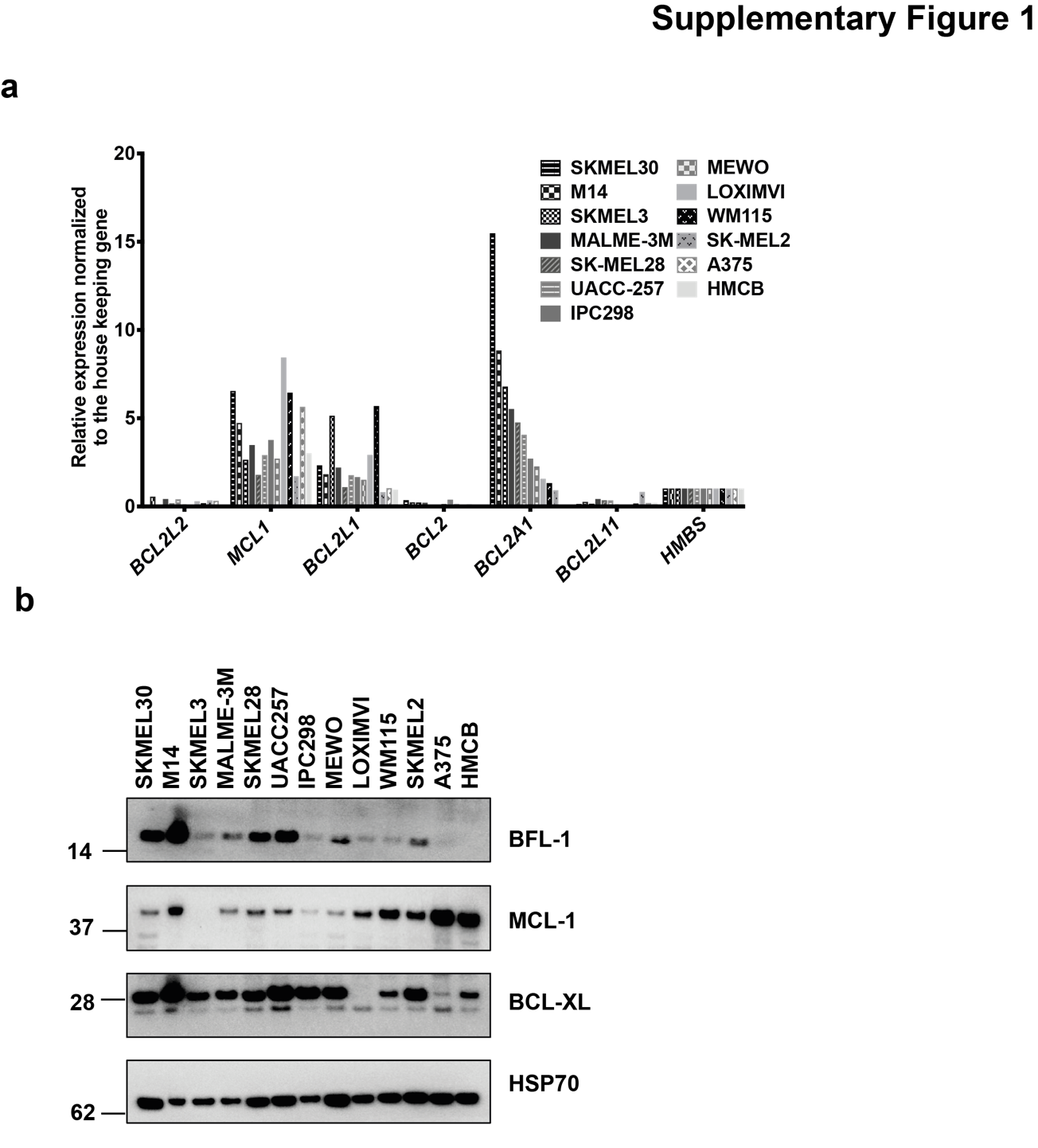


**Supplementary Figure 1. Expression of pro-survival BCL-2 family proteins in melanoma cell lines.** Western blot analysis was performed to determine the expression of BFL-1, MCL-1 and BCL-XL proteins in the indicated human melanoma derived cell ines. Probing for HSP70 was used as a protein loading control.


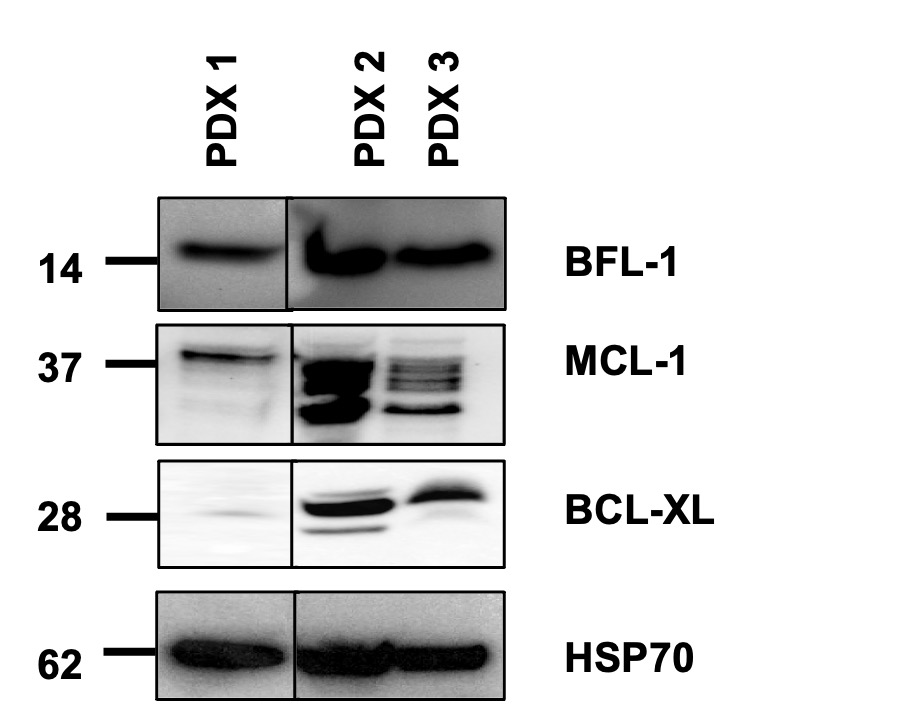


**Supplementary Figure 2. Expression of pro-survival proteins in human melanoma patient derived xenografts (PDX).** Western blot analysis was performed to determine the expression of BFL-1, MCL-1 and BCL-XL proteins in the indicated PDXs. Probing for HSP70 was used as a protein loading control. PDX1 has *NRAS^Q61K^* mutation and PDX 2 and 3 has *BRAF^S467L^* mutation. Lysates were prepared from snap frozen tissues from tumours formed in NGS mice.


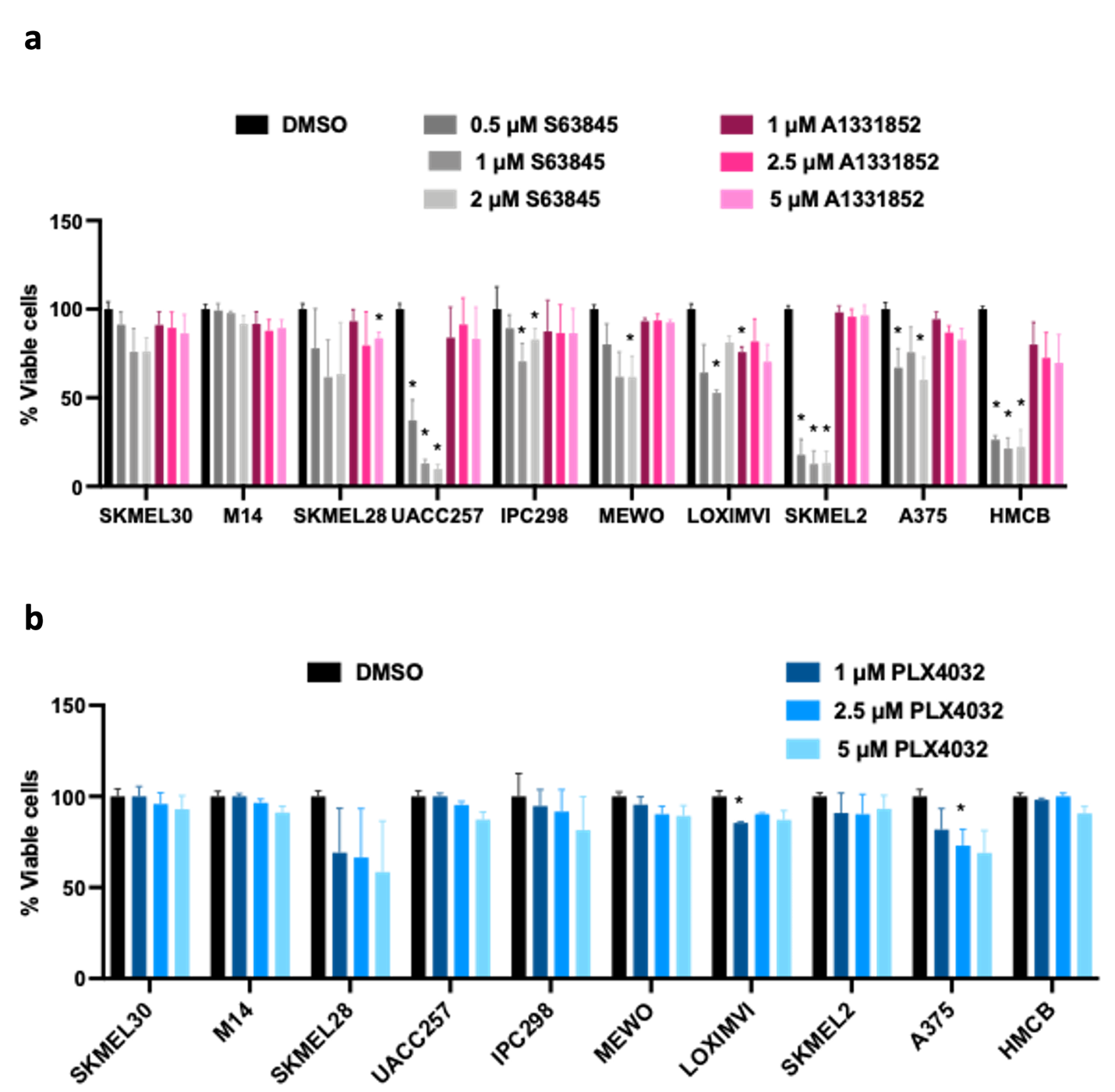


**Supplementary Figure 3. Analysis of the responses of human melanoma derived cell lines to treatment with different BH3 mimetic drugs or a mutant BRAF inhibitor, used as single agents.** **(a)** Single anti-cancer agent treatments of the indicated human melanoma cell lines for 72 h with an MCL-1 inhibitor (S63845) or a BCL-XL inhibitor (A1331852) or **(b)** a mutant BRAF inhibitor (PLX4032). Data represents mean ±SEM of three independent experiments. * P < 0.05 . DMSO was used as the vehicle control.


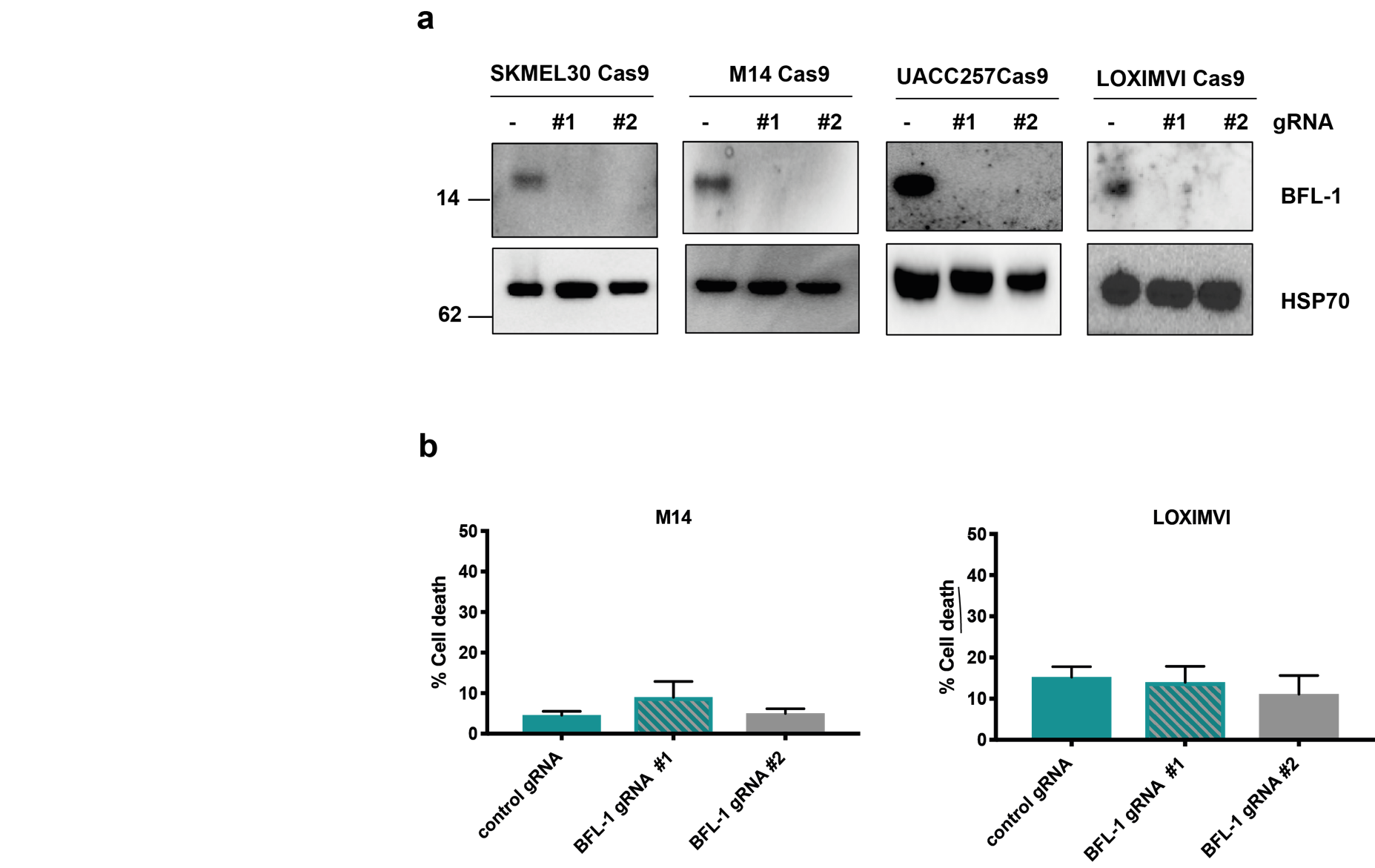


**Supplementary Figure 4.** **Generation of BFL-1 knockout human melanoma cell lines using CRISPR/Cas9 and testing of spontaneous cell death. (a)** Western blot analysis of the BFL-1 protein levels in the indicated human melanoma cell lines expressing Cas9 that had also been transduced with two distinct guide RNAs (sgRNA) targeting the human *BFL1* gene or a control sgRNA (targeting the mouse *Bim* gene) and treated for 3 days with with 1 μg/mL doxycyclin to induce sgRNA expression for 3 days. **(b)** The indicated human melanoma cell lines transduced either with a doxycyclin-inducible control sgRNA or either of two distinct dox-inducible sgRNAs targeting the human *BFL1* gene were treated for 72 h with doxycycline and then their survival was determined by staining with Annexin V-AF647 plus DAPI staining followed by FACS analysis.

**Methods**

**Compounds**

The mutant BRAF inhibitor PLX4032 (#S1267), the MEK1/2 inhibitor Trametinib (#S2673) and the ERK inhibitor Ulixertinib (#S7854) were purchased from Selleck-chem. The MCL-1 inhibitor S63845 (#A-6044) was purchased from ActiveBiochem. The BCL-XL inhibitor A-1331852 was kindly supplied by Prof Guillaume Lessene (WEHI).

**Cell culture**

The human melanoma derived cell lines were cultured in RPMI-1640 medium (Life Technologies) supplemented with 10% foetal bovine serum and 100 U/mL penicillin/streptomycin at 37 °C in a humidified incubator with 5% CO_2_.

**cDNA synthesis and qPCR analysis**

RNA was extracted from cells using TRIzol reagent (#15596026, Invitrogen). One μg total RNA from each sample was used for cDNA synthesis using Superscript III First Strand Synthesis Supermix (#18080400, Invitrogen) following kit instructions. Quantitative RT-PCR analysis was performed using TaqMan Gene Expression Assay (FAM) and TaqMan Fast Advance Master Mix (#4444963, Invitrogen). The RT-PCR reactions were performed using Applied Biosystems Viia7 Real-Time PCR System. All RT-PCR reactions were performed in triplicate. Relative quantification was performed using the ΔCT method. Relative expression levels of each gene of interest were normalised to the house keeping gene hydroxymethylbilane synthase (HMBS).

**FACS-based apoptosis assay**

Cells (10,000 per well) were seeded in a 96-well plate and 24 h later treated for 72 h with drugs as single agents or in the indicated combinations. For all experiments, cell survival was determined by resuspending cells in Annexin V–binding buffer (0.1 M HEPES pH 7.4, 1.4 M NaCl, 25 mM CaCl_2_) containing Annexin V-AF647 (#A23204, Life Technologies) and DAPI (2 μg/mL) followed by flow cytometric analysis using a LSR II flow cytometer (BD Biosciences). Data were analysed using FlowJo software (FlowJo LLC). The percentages of viable cells (Annexin V negative/DAPI negative) were presented relative to the percentages of viable cells from control cultures containing vehicle (DMSO).

**Western blot analysis**

Total protein extracts were prepared by lysing cells in lysis buffer (20 mM Tris-pH 7.4, 135 mM NaCl, 1.5 mM MgCl_2_, 1 mM EGTA, 10% (v/v) glycerol and 1% (v/v) Triton-X-100; Sigma-Aldrich) with complete protease inhibitor cocktail (Roche) for 1 h at 4 °C. Equal amounts of protein were electrophoresed on NuPAGE 4–12% Bis Tris gels (Invitrogen) before transferring to nitrocellulose membranes (Life Technologies) and probing with primary antibodies: monoclonal rat anti-MCL-1 (clone 19C4-15, WEHI antibody facility), monoclonal rat anti-BCL-XL (clone 9C9, WEHI antibody facility), monoclonal mouse anti-BFL-1 clone 6A7, WEHI antibody facility) ^18^ and monoclonal-mouse anti-HSP70 (clone N6, W. Welch USCF). Secondary anti-rat (#3010-05) IgG or anti-mouse IgG antibodies (#1010-05) conjugated to HRP (Southern BioTech, Birmingham, AL, USA) were applied, followed by addition of the Luminata Forte Western HRP substrate (Millipore, Billerica, MA, USA) for visualisation of protein bands. Membranes were imaged using the ChemiDoc XRS+ machine with ImageLab software (Bio-Rad). Quantification of the Western blot band intensities was carried out using the Fiji Image J software.

**CRISPR/Cas-9 deletion of the human *BFL-1* gene**

Lentiviral particle production for the constitutive expression of Cas9 (with mCherry as a marker) or doxycycline-inducible expression of sgRNAs (with constitutive expression of GFP as a marker) was performed as described previously ^13^. Virus-containing supernatants were used to infect cells with Cas9-expressing lentivirus. mCherry^+^ cells were sorted using a BD FACSAria III flow cytometer, and subsequently infected with lentivirus for doxycycline-inducible expression of a sgRNA. The mCherry^+^/GFP^+^were then sorted using a BD FACSAria III flow cytometer. The expression of the sgRNAs was induced by treatment with doxycycline (1 μg/mL, Sigma) for at least 3 days prior to Western blot analysis to determine the efficiency of gene deletion, and treatment with BH3-mimetic drugs or other anti-cancer drugs, either as single agents or in the indicated combinations of drugs.

**Statistical analysis**

Prism software (GraphPad) was used to generate graphs. Data are presented as the mean ± S.E.M. P values were calculated by performed Two-way ANOVA followed by multiple comparisons testing. *P* values < 0.05 were considered as statistically significant, and *P* values > 0.05 were considered non-significant.
