# Supplementary material for: Removal of BFL-1 sensitises some melanoma cells to killing by BH3 mimetic drugs
Source: Cell Death Dis. 2022 Apr 4;13(4):301. doi: 10.1038/s41419-022-04776-y (PMC8980089; doi:10.1038/s41419-022-04776-y)
Supplement: Supplementary file 3 — Point by Point Reply [file 41419_2022_4776_MOESM3_ESM.docx]

**Point by point rebuttal:**

We thank the reviewers for their overall constructive feedback for the improvement of the manuscript. Further, we have addressed the points raised by the reviewers.

**Reviewer Comments:** 

**Referee #1 (Remarks to the Author):**

Gangoda et al. submitted this commentary in which they studied the consequences of BFL-1 loss on responsiveness to BH3 mimetic drugs and kinase inhibitors relevant in the treatment of melanoma. Ultimately, they show that one cell line responds properly to loss of BFL-1, combined with BCL-XL and MCL-1 inhibition. Overall, the data is purely descriptive and not of high interest, and therefore not suitable for this journal. Please see a number of comments below that might assist in further work and presentation of the data. 

1. The title overstates the findings. Only one cell line out of a panel of 13 was convincingly shown to be sensitized by BFL-1 loss.

*We agree with the reviewer and have now changed the title to indicating that BFL-1 loss only makes some melanoma cells sensitive to BH3 mimetics.*

2. First sentence: Obviously, the authors must have made a mistake in stating a 20% 5-year survival and linking this statement to a reference from 1996. Things have moved on massively in melanoma. Please correct.

*The 5-year survival for metastatic melanoma has not changed significantly over the past couple of decades. However, we have now included a more recent citation.*

3. Line 27. This sentence needs to consider actual current treatment (combinations) and outcomes. Single-drug BRAF mutant inhibition is not the clinical state of the art.

*We thank the reviewer for this comment. We have changed the sentence to reflect that mutant BRAF inhibitors are used in combination with other chemotherapy drugs.*

4. The authors claim BCL-2 is not expressed in reference to FigS1 but do not show data supporting this claim. In fact, BCL-2 might instead confer part of the high resistance in these cells.

*We did not test BCL-2 inhibition alone or in combination with mutant BRAF inhibitors, as it has been shown before that this has no effect on the survival of these cells. We have mentioned this now in the manuscript with the relevant reference.*

5. Line 43: The statement on survival in relation to the BRAF inhibitor is misleading. Viability was measured after 72 h and not cell death. The lower reading for BRAF inhibitor treatment might stem simply from attenuated proliferation of the cells rather than the cells dying.

*It was indeed the cell death that was measured by staining with Annexin-V-AF647 and DAPI after 72h followed by flow cytometry. However, we represented the Annexin-V and DAPI negative population data as viable cells in the graph while excluding Annexin V single positive, DAPI single positive and Annexin-V and DAPI double positive cells as dead cells.*

6. Line 46: Typo to be corrected

*We have re-checked the manuscript for typos.*

7. Line 51: The authors state that LOXIMVI cells express high amounts of BFL-1. They do not.

*We have changed the wording to say LOXIMVI cells show medium levels of BFL-1 expression.*

8. Line 58: The statement that BRAF inhibition does not further enhance death is trivial and a twisted view of the data. If practically all cells die by combined BCL-XL/MCL-1 inhibition, it is impossible to measure BRAF inhibitor effects. This would require titrations to lower inhibitor concentrations.

*We thank the reviewers for this perspective. We agree with this comment and have removed this statement from the manuscript.*

9. Line 62: This statement is guess work

*We have removed the sentence describing the difference in cell death between two different guide RNA transduced cell populations.*

10. Line 77: This sentence is masking that this was observed convincingly only in one single cell line.

*We have added a sentence to reflect that this was only convincingly shown in one out of four cell lines tested.*

11. Since multiple comparisons were made, a simple t-test is inappropriate to judge on statistically significant differences between the groups analysed.

*We have now performed Two-way ANOVA followed by multiple comparisons testing to calculate P values.*

12. Fig1 would benefit from using the same colour for the gRNA groups and making the labelling bigger. The drug treatments might be easier readable if the combinations are indicated by a +/- table style below the bar graphs.

*We have changed the graphs so that each gRNA group is the same colour and have made the labelling bigger. But we have kept the drug treatment as text describers.*

13. Fig S3: Typo in panel A for 1 µM S63845

*We thank the reviewers for pointing this out. We have corrected this error.*

**Referee #2 (Remarks to the Author):**

The study by Gangoda et al. claims removal of BFL-1 to sensitise melanoma cells to killing by BH3 mimetic drugs. In the course of experiments, however, it turns out that BFL-1 knock out only sensitsed “certain”, namely 2 out of 4 (M14, SKMEL30) but tentatively also desensitized 2 out of 4 (UACC, LOXIMVI) cells lines to combined MCL-1/BCL-XL treatment. Based on these heterogeneous data, the authors conclude that for efficient melanoma cell killing all three BCL-2 proteins (BFL-1, MCL-1 and BCL-XL) must be inhibited.

Major: 

1. To date no specific BFL-1 inhibitor is available. Therefore, the impact of BFL-1 inhibition to melanoma treatment remains illusive. Recently, CDK9 inhibition was shown to cause concomitant downregulation of MCL-1 and BFL-1 (Boiko et al., Blood (2021) 137 (21): 2947–2957), and might have been a good option for more detailed investigations.

*We have explored if CDK9 inhibitor (AZ5576) is capable of reducing BFL-1 levels in melanoma cells. M14 melanoma cells were treated for 6 h with various concentrations of AZ5576 and cells subjected to Western blot analysis. Although CDK9 inhibitors did lower the MCL-levels in cells unfortunately it did not have any effect on BFL-1 levels in the M14 melanoma cells. We have shown this data below. Hence, we did not include the CDK9 inhibition in the manuscript as this interesting observation forms part of another larger study in our laboratory.*

2. Even though the authors only refer to BRAF mutated melanoma in the introduction, 6 out of the 13 cell lines initially investigated regarding expression of BFL-1, MCL_1 and BCL-XL harbor NRAS mutations (M14, IPC298, SKMEL2, HMCB), are wildtype for both, NRAS/BRAF mutation (MEWO) or are heterozygous for BRAF mutation (LOXIMVI).

*It has been well established that M14 melanoma cells carry BRAF ^V600E^ mutation and is wild type for NRAS. However, we agree with the reviewer IPC298, SKMEL2, HMCB are NRAS mutant, MEWO is wild type for both NRAS/BRAF and that LOXIMVI is heterozygous for BRAF mutation. Three of the four cell lines which we chose to knockout BFL-1 in, harbour BRAF mutations (M14, UACC257 and LOXIMVI) and SKMEL30 has a NRAS mutation. We tested all the melanoma cell lines we had access to for cell survival protein expression despite their mutational status.*

3. It remains unclear why LOXIMVI cells were selected for BFL-1 knock down experiments, because these cells initially express only low BFL-1 level. Moreover, BFL-1 expression in primary cells must be investigated as well.

*We only tested LOXIMVI as an additional control for a medium BFL-1 expressing cell line. Deletion of BFL-1 in LOXIMVI had almost no effect on its drug response. We had very limited access to primary melanoma cells from PDX tissues. We were unable to expand them in culture to perform knockout studies despite multiple attempts.*

4. Essential controls on the cytotoxicity of the drug combination on primary cells, or the xenograft models are missing.

*We had limited access to primary cells. Hence, we were unable to perform knockout studies or drug treatment for these*.

Minor: 

1. The manuscript still contains a number of typos.

*We have proofread the manuscript once more for typos.*
